# Three-dimensional spheroid cell culture of umbilical cord tissue-derived mesenchymal stromal cells leads to enhanced paracrine induction of wound healing

**DOI:** 10.1186/s13287-015-0082-5

**Published:** 2015-05-09

**Authors:** Jorge M Santos, Sérgio P Camões, Elysse Filipe, Madalena Cipriano, Rita N Barcia, Mariana Filipe, Mariana Teixeira, Sandra Simões, Manuela Gaspar, Diogo Mosqueira, Diana S Nascimento, Perpétua Pinto-do-Ó, Pedro Cruz, Helder Cruz, Matilde Castro, Joana P Miranda

**Affiliations:** ECBio – Investigação e Desenvolvimento em Biotecnologia S.A., Rua Henrique Paiva Couceiro, N° 27, 2700-451 Amadora, Portugal; iMed.ULisboa – Research Institute for Medicines, Faculty of Pharmacy, Universidade de Lisboa, Av. Prof. Gama Pinto, 1649-003 Lisbon, Portugal; I3S – Instituto de Investigação e Inovação em Saúde, Universidade do Porto, Porto, Portugal; INEB – Instituto de Engenharia Biomédica, Universidade do Porto, Rua do Campo Alegre, N° 823, 4150-180 Porto, Portugal; ICBAS – Instituto de Ciências Biomédicas Abel Salazar, Universidade do Porto, Rua de Jorge Viterbo Ferreira, N° 228, 4050-313 Porto, Portugal; Unit for Lymphopoiesis, Immunology Department, INSERM U668, University Paris Diderot, Sorbonne Paris Cité, Cellule Pasteur, Institut Pasteur, Paris, 75015 France

## Abstract

**Introduction:**

The secretion of trophic factors by mesenchymal stromal cells has gained increased interest given the benefits it may bring to the treatment of a variety of traumatic injuries such as skin wounds. Herein, we report on a three-dimensional culture-based method to improve the paracrine activity of a specific population of umbilical cord tissue-derived mesenchymal stromal cells (UCX®) towards the application of conditioned medium for the treatment of cutaneous wounds.

**Methods:**

A UCX® three-dimensional culture model was developed and characterized with respect to spheroid formation, cell phenotype and cell viability. The secretion by UCX® spheroids of extracellular matrix proteins and trophic factors involved in the wound-healing process was analysed. The skin regenerative potential of UCX® three-dimensional culture-derived conditioned medium (CM3D) was also assessed *in vitro* and *in vivo* against UCX® two-dimensional culture-derived conditioned medium (CM2D) using scratch and tubulogenesis assays and a rat wound splinting model, respectively.

**Results:**

UCX® spheroids kept in our three-dimensional system remained viable and multipotent and secreted considerable amounts of vascular endothelial growth factor A, which was undetected in two-dimensional cultures, and higher amounts of matrix metalloproteinase-2, matrix metalloproteinase-9, hepatocyte growth factor, transforming growth factor β1, granulocyte-colony stimulating factor, fibroblast growth factor 2 and interleukin-6, when compared to CM2D. Furthermore, CM3D significantly enhanced elastin production and migration of keratinocytes and fibroblasts *in vitro*. In turn, tubulogenesis assays revealed increased capillary maturation in the presence of CM3D, as seen by a significant increase in capillary thickness and length when compared to CM2D, and increased branching points and capillary number when compared to basal medium. Finally, CM3D-treated wounds presented signs of faster and better resolution when compared to untreated and CM2D-treated wounds *in vivo*. Although CM2D proved to be beneficial, CM3D-treated wounds revealed a completely regenerated tissue by day 14 after excisions, with a more mature vascular system already showing glands and hair follicles.

**Conclusions:**

This work unravels an important alternative to the use of cells in the final formulation of advanced therapy medicinal products by providing a proof of concept that a reproducible system for the production of UCX®-conditioned medium can be used to prime a secretome for eventual clinical applications.

**Electronic supplementary material:**

The online version of this article (doi:10.1186/s13287-015-0082-5) contains supplementary material, which is available to authorized users.

## Introduction

Most multipotent mesenchymal stromal cells (MSCs) are capable of immune evasion and display immune-suppressive properties, thereby providing an allogeneic, ready-to-use, off-the-shelf cell product option for therapeutic applications [1,2]. Thus, the beneficial effect of MSCs for the treatment of a variety of traumatic injuries such as open wounds has been extensively explored [3-6]. It was originally assumed that the observed benefits were the result of cellular engraftment and differentiation towards replacement of injured cells. However, several reports demonstrate that it rather results from a positive action on the tissue remodelling process via the paracrine secretion of trophic factors, such as cytokines, and/or cell-to-cell chemotactic interactions that modulate inflammation, immune reactions and activity of surrounding cells [1,5,7-9]. Most of these data have been obtained with bone marrow-derived MSCs (BM-MSCs) [6,10], while increasing evidence shows that MSCs from other sources could have distinct characteristics with regards to differentiation, expansion potential with concomitant genomic stability, and tissue regeneration capabilities [2,6,11].

Amongst the MSCs producing promising results in on-going pre-clinical trials are the umbilical cord tissue-derived MSCs, UCX® [12,13]. UCX® are isolated, expanded and cryopreserved according to a patented method (PCT/IB2008/054067; WO 2009044379) designed to produce a highly homogeneous population of cells that comply with the MSC standards as defined by the International Society for Cellular Therapy [14]. Recently, the UCX® tissue regeneration capacity has been functionally demonstrated in several animal models for myocardial infarction and rheumatoid arthritis [2,13]. Furthermore, our *in vitro* studies, performed with conditioned medium (CM) produced by UCX® grown in classical two-dimensional monolayer cultures, have demonstrated the potential for promoting cutaneous wound healing [12]. Namely, UCX® were shown to be strongly motogenic towards keratinocytes and to be able to attract BM-MSCs *in vivo*, in a one-way specific granulocyte-colony stimulating factor (G-CSF)-mediated mechanism [12]. These results have suggested positive UCX® implications in the early stages of wound healing as well as in the proliferation and remodelling stages, through potential recruitment of circulating CD34^−^ CD45^−^ cells that are known to promote fibroblast migration, extracellular matrix (ECM) production, angiogenesis and vasculogenesis [12]. More recently and supporting our *in vitro* evidence, umbilical cord Wharton’s jelly-derived MSCs (WJ-MSCs) have been shown to consistently improve the healing response in mouse models of dermal repair [15-17].

Routinely, MSCs are expanded and maintained in traditional monolayer (two-dimensional) cultures where cells migrate and proliferate while adhering to the plastic surface of static culture flasks. In addition, two-dimensional systems consist of growth conditions that are further away from the *in vivo* physiological environment, since they lack three-dimensional cell-to-cell interactions. The MSC phenotypes resulting from two-dimensional culture systems are therefore more limited in benefits that a more matrix-like environment may bring. In an attempt to recreate the complex microenvironment of living systems, the use of MSC three-dimensional *in vitro* culture models has gained increasing attention [1,18-22], namely as a procedure for enhancing chondrogenic differentiation [23] or for improving the therapeutic potential of cells [1,19]. Recently, Sabapathy and colleagues [24] found that WJ-MSCs seeded on decellularized amniotic membrane scaffolds proved to have higher wound-healing capabilities when transplanted onto skin injuries of SCID mice model than WJ-MSCs alone, showing that a three-dimensional environment can prime WJ-MSCs to a more therapy-driven phenotype. Alternatively, a less complex three-dimensional model is the spinner flask suspension culture (SFSC), where cells self-assemble into spheroid-like structures, thus enabling greater cell-cell and cell-matrix interactions [19-22,25-27]. The SFSC is also amenable for both cell expansion and differentiation [28], as well as for up-scaling processes avoiding some regulatory constraints related to adhering supports and scaffolds. In this work, we aimed at testing the hypothesis that the natural self-aggregation of UCX® is an effective system for priming these cells towards a paracrine activity that would further promote wound healing. For this purpose, a reproducible and scalable three-dimensional culture system using SFSC for extended maintenance of multipotent UCX® spheroids was developed, devoid of supporting matrices or the use of complex scaffolds. The environment within UCX® spheroids successfully mimicked the native cell microenvironment resulting in a richer secretome profile. Indeed, our comparative analysis showed that the resulting three-dimensional conditioned medium (CM3D) improved wound healing both *in vitro* and *in vivo* when compared to two-dimensional conditioned medium (CM2D). In summary, our three-dimensional culture model may represent an alternative system to augment the UCX®-driven potential to improve the regenerative response of human skin to injury. The scalability of this system further represents a new approach for the eventual production of UCX®-CM for therapeutic purposes, avoiding the use of cells in the final medicinal product.

## Materials and methods

### Ethics and regulations

This study was approved by the Ethics Committee of the Hospital Dr. José de Almeida (Cascais, Portugal), in the scope of a research protocol between ECBio (Research & Development in Biotechnology, S.A.) and HPP Saúde (Parcerias Cascais, S.A.). Umbilical cord donations, with written informed consents, as well as umbilical cord procurement, were made according to Directive 2004/23/EC of the European Parliament and of the Council of 31 March 2004 on setting standards of quality and safety for the donation, procurements, testing, processing, preservation, storage and distribution of human tissues and cells. All animal experiments were carried out with the permission of the local animal ethical committee in accordance with the EU Directive (2010/63/EU), Portuguese law (DL 113/2013) and all relevant legislations. The experimental protocol was approved by Direcção Geral de Alimentação e Veterinária.

### Cell culture reagents

Cell culture media and supplements used in this work were all purchased from Sigma-Aldrich (Madrid, Spain), unless stated otherwise. Foetal bovine serum (FBS) and Trypsin/ethylenediamine tetraacetic acid (EDTA) were obtained from Gibco (Life Technologies, Madrid, Spain).

### UCX® isolation and culture

UCX® were isolated from umbilical cords of healthy newborn babies (57% male) upon informed consent of healthy parturient mothers, according to Santos and colleagues [2,29]. Briefly, fresh human umbilical cords were obtained after full-term natural births, transported to the laboratory facilities in a sterile container containing saline buffer and processed within a period up to 48 hours. Identical cord tissue sections were digested, using a precise ratio between tissue mass, tissue digestion enzyme activity units, digestion solution volume and void volume using collagenase. The procedure includes three recovery phases in order to avoid non-conformities related to percent cell recovery. In the first cell recovery phase, cells dissociated from the tissue are recovered by a static horizontal incubation. The remaining cells are incubated in a static monolayer culture in UCX® culture medium (α-modified Eagle’s medium (MEM) with 2 mM L-glutamine, 1 g/L glucose, 2.2 g/L sodium bicarbonate) supplemented with 20% FBS, at 37°C in 5% CO_2_ humidified atmosphere, with medium change every 72 hours until they reached around 80% confluence. Cells were cryopreserved in 10% dimethyl sulphoxide (DMSO) stock solution and 20% FBS, using control rate temperature decrease. When necessary UCX® cryopreserved at passages between passage (P)3 and P5 were thawed and further expanded during a maximum of 30 cumulative population doublings (cPDs), corresponding to P12 in culture. The range of cPDs chosen allowed for enough expansion for eventual production of large cell numbers and amounts of CM but keeping cPDs within the genomic stability range. UCX® are known to undergo at least 55 cPDs (P22) before reaching senescence and keeping all MSC traits.

In two-dimensional (static monolayer) cultures, cells were seeded at a density of 1 × 10^4^ cells/cm^2^ in UCX® medium supplemented with 10% FBS and incubated at 37°C in a humidified atmosphere with 5% CO_2_. Cell passage was performed by Trypsin/EDTA 0.05% incubation for 5 minutes every 72 hours.

In three-dimensional (SFSC) cultures, 125 mL spinner vessels with ball impeller containing UCX® medium supplemented with 10% FBS were inoculated with single-cell suspensions obtained from two-dimensional cultures at a concentration of 1 × 10^6^ cells/mL. To promote cell aggregation, the spinner vessels were agitated at 80 rpm and kept at 37°C in a humidified atmosphere of 5% CO_2_. After 24 hours, 50% of cell culture supernatant was changed with fresh UCX® medium supplemented with 10% FBS (v/v). Culture medium was replaced every 3 to 4 days to guarantee nutrient availability and to decrease the accumulation of toxic by-products. The stirring rate was adjusted to 110 rpm in order to keep spheroid size below 350 μm.

For spheroid cell plating back into two-dimensional cultures, a 5 mL cell suspension from three-dimensional cultures was collected at day 7. Spheroids were digested with 0.25% Trypsin/EDTA for 15 minutes resulting in smaller spheroids that were inoculated in a six-well plate. Cells were allowed to adhere in monolayer and proliferate for 1 passage. Cells were then collected for flow cytometry analysis of cell surface marker expression, and assessment of tri-lineage differentiation potential as described below.

### UCX® characterization

#### Flow cytometry

Cell surface marker expression was analysed by flow cytometry. For the characterization of UCX® in both two-dimensional and three-dimensional cultures, both cell detachment from culture flasks and dissociation from spheroids were achieved by using 0.25% Trypsin/EDTA and the resulting single cell suspension washed with 2% bovine serum albumin (BSA) in phosphate-buffered saline (PBS). Detection of cell surface markers was performed with the following antibodies and their respective isotypes after incubation for 1 hour at 4°C (all from BioLegend (San Diego, CA, USA) unless stated otherwise): phycoerythrin (PE) anti-human CD105 (eBioScience, San Diego, CA, USA); APC anti-human CD73; PE anti-human CD90; APC anti-human CD44; PerCP/Cy5.5 anti-human CD45; fluorescein isothiocyanate (FITC) anti-human CD34; FITC anti-human CD31; PerCP/Cy5.5 anti-human CD14; Pacific Blue anti-human CD19 and pacific-blue anti-human HLA-DR. All samples were acquired on a Gallios (Beckman Coulter, Pasadena, CA, USA) and the results analysed with Kaluza software (Beckman Coulter). A minimum of 1 × 10^4^ events were acquired per surface marker. One replicate was analysed per independent experiment (n = 4).

#### Tri-lineage differentiation

Tri-lineage differentiation was performed in UCX® two-dimensional and three-dimensional cultures. Spheroids were dissociated into a single cell suspension with 0.25% Trypsin/EDTA and transferred to the appropriate culture plates for cell proliferation and expansion. Adipogenic and osteogenic differentiation was performed based on Lee and colleagues [30] and were initiated when cultures reached 80 to 100% confluence. For the chondrogenic differentiation [31], non-dissociated spheroids were also used for differentiation.

To induce adipogenic differentiation, UCX® were incubated in UCX® medium supplemented with 20% FBS, 10 μg/mL insulin, 200 μM indomethacin, 0.5 mM isobutylmetylxantine, and 1 μM dexamethasone for 3 days and 1 day in UCX® medium supplemented with 20% FBS and 10 μg/mL insulin. The medium was changed every 3 days for a period of 21 days. To induce osteogenic differentiation, cells were incubated in UCX® medium supplemented with 10% FBS, 10 mM β-glycerol phosphate, 100 nM dexamethasone and 50 μg/mL ascorbate-2-phosphate. The medium was changed every 3 days during 21 days. Finally, for chondrogenic differentiation, cells (both dissociated cells and spheroids) were maintained in suspension as pellets, incubated with Dulbecco’s modified Eagle’s medium (DMEM) with 4 mM glutamine and 1 g/L D-(+)-glucose, supplemented with 1% FBS, 6.25 μg/mL insulin, 10 ng/mL transforming growth factor (TGF)-β1 (Tebu-bio, Le-Parray-en-Yvelines, France), and 50 μM ascorbate-2-phosphate. The medium was changed every 3 days during 21 days. Cytochemical staining of cells was performed as described by Wang and colleagues [32]. Briefly, cells were fixed with paraformaldehyde 4% for 20 minutes. In adipogenic and osteogenic differentiation protocols, cells were stained with Oil Red O for 10 minutes and alkaline phosphatase for 30 minutes, respectively. For chondrogenic differentiation, the chondrospheres were fixed in formalin, embedded in paraffin and cut into sections of 5 μm and stained with alcian blue for 30 minutes. The presence of stained cells was confirmed by inverted microscopy with phase contrast (Leica, DMIL HC, Wetzlar, Germany).

### UCX® viability evaluation

#### Protein quantification

Culture biomass within two-dimensional and three-dimensional cultures was evaluated by quantification of total protein using the BCA protein assay kit (Novagen, San Diego, CA, USA), after lysis of the cell pellet with 0.1 M NaOH at 37°C for 24 hours. A linear calibration curve to relate total protein with cell number was generated to further estimate UCX® cell number.

#### Cell membrane integrity assay

The qualitative assessment of the cell plasma membrane integrity during culture was performed using the enzyme substrate fluorescein diacetate (Sigma-Aldrich) and the DNA-dye propidium iodide (Sigma-Aldrich). Briefly, cell aggregates were incubated with 20 μg/mL fluorescein diacetate and 1 μg/mL propidium iodide in PBS for 2 to 5 minutes and then observed using an inverted fluorescence microscope (Nikon Eclipse Ti-U, Tokyo, Japan).

#### UCX® spheroid visualization and measurement

UCX® spheroids were observed by bright field microscopy (Olympus CK30, Olympus, Tokyo, Japan), and their average diameter determined by a geometric mean of three diameters per spheroid as described previously, using the following equation: average diameter = (d1 × d2 × d3)^1/3^ [33]. Spheroid diameters were measured using Motic Images Version 2.0 software (Xiamen, China).

#### Haematoxylin and eosin staining

UCX® spheroids were resuspended in Tissue Tek® O.C.T.™ (Sakura, Zoeterwoede, The Netherlands) for preparing cryosections of 10 μm. Slides were first stained with Harris’s haematoxylin (Sigma-Aldrich) for 10 minutes, followed by an incubation step with HCl 1% (v/v) in 70% EtOH, and by Eosin Y (Sigma-Aldrich) staining for 2 minutes. Slides were then submitted to increasing concentrations of ethanol and finally incubated in xylene (EMD Chemicals, Inc., Gibbstown, NJ, USA). Samples were mounted with Entellan® (Merck, Darmstadt, Germany). Images were acquired on an Olympus CK30 inverted microscope and processed using Motic Images Version 2.0 software.

#### Immunofluorescence microscopy

Cryosections from UCX® spheroids were prepared as described above. Tissue sections were fixed with cold acetone for 10 minutes and blocked for 1 hour with 4% FBS and 1% BSA in PBS. Incubation with primary antibody was performed in a humified chamber for 2 hours at room temperature or overnight at 4°C. The primary antibodies used were: Ki67 (Rabbit IgG, AB16667, Abcam, Cambridge, UK), diluted 1:100; Collagen IV (Goat, AB769, Millipore, Billerica, MA, USA) diluted 1:10; Fibronectin (Rabbit, F-3648, Sigma-Aldrich) diluted 1:400; Laminin (Rabbit IgG, L9393, Sigma-Aldrich) diluted 1:50; collagen I (Rabbit IgG, AB21285, Abcam) diluted 1:200. All primary antibodies were diluted in 1% BSA in PBS. The incubation with secondary antibodies was carried out for 1 hour at room temperature. The secondary antibodies used were as follows: goat anti-rabbit 594 (1:500) for Ki67 staining; rabbit anti-goat-biotin (1:400) followed by streptavidin 555 (1:500) for Collagen IV staining; donkey anti-rabbit IgG 568 (1:1000) for Laminin staining; donkey anti-rabbit IgG 647 (1:1000) for Collagen I staining and goat anti-rabbit 488 (1:1000) for Fibronectin staining. All secondary antibodies were purchased from Invitrogen (Carlsbad, CA, USA). Sections were mounted using ProLong gold antifade with DAPI (Invitrogen) or Fluoroshield™ with DAPI (Sigma-Aldrich), and observed on an inverted fluorescence microscope (Axiovert 200 M, Carl Zeiss, Oberkochen, Germany) coupled with a monochrome camera (AxioCam MNC, Carl Zeiss).

### Conditioned media preparation and evaluation

#### Conditioned media production

CM was produced from cells having undergone the same number of cPDs.

UCX® CM3D was obtained by inoculation of cells as described above, but subjected to successive medium adaptation replacements in order to reduce FBS concentration to 0%. At day 2 of culture, FBS concentration was reduced to 5%. After 3 days, medium was changed with UCX® medium without FBS and medium volume adjusted accordingly, in order to obtain a conditioning volume per cell as in the two-dimensional system. After 48 hours of conditioning, CM3D was collected under sterile conditions.

For the production of UCX® CM2D, 1.75 × 10^6^ cells were seeded in 175 cm^2^ t-flasks and kept in UCX® medium supplemented with 5% FBS until they reached 90% confluence. At this stage, cells were carefully washed with fresh α-MEM and medium was replaced by α-MEM without FBS, at a final volume of 25 mL. After conditioning for 48 hours, CM2D was harvested under sterile conditions.

The control sample consisted of UCX® medium which was never in contact with cells. CM3D, CM2D and control were concentrated 10× using 3-kDa cut-off spin concentrators (Pall, Ann Arbor, MI, USA). Total protein content of CM2D, CM3D and controls was quantified using the BCA protein assay kit (Novagen) according to the manufacturer’s instructions. Samples were stored at −80°C until further use.

#### Methylthiazolyldiphenyl-tetrazolium bromide viability assay

The cytotoxicity of CM2D and CM3D was evaluated by the methylthiazolyldiphenyl-tetrazolium bromide (MTT) reduction assay on two cell types of cutaneous origin: primary human dermal fibroblasts (HDF; ATCC cat: PCS-201-012, Middlesex, UK), and the spontaneously immortalized keratinocyte cell line (HaCaT; Cell-Line-Service cat: 300493, Eppelheim, Germany). HDF and HaCaT were seeded in 96-well plates at a density of 1.5 × 10^4^ cells/cm^2^ and 4.0 × 10^4^ cells/cm^2^, respectively, in DMEM with 4 g/L D-(+)-Glucose supplemented with 10% FBS in a humidified chamber at 37°C in a 5% CO_2_ atmosphere. After 24 hours of incubation, cell culture medium was replaced by CM2D or CM3D 0.5×, 1×, 3×, 6× and 10× concentrated. Cells were also incubated with 200 μL complete cell culture medium and DMSO 20% in α-MEM as a positive control and control of death, respectively. After 48 hours, cells were carefully washed with 100 μL PBS, and 200 μL 0.5 mg/mL MTT (Sigma-Aldrich) in complete cell culture medium was added. HDF were incubated for 3 hours and HaCaT for 45 minutes, both in a humidified chamber at 37°C in a 5% CO_2_ atmosphere. The purple crystals were solubilized with 200 μL DMSO and measured at 570 nm using a microplate spectrophotometer (SPECTROstar Omega; BMG LabTech, Ortengerg, Germany). Results were expressed as a percentage relative to the positive control. Four wells were used for each sample, and three independent experiments were performed.

#### Elastin quantification

Elastin was quantified in HDF and HaCaT cells seeded in 12-well plates. At a confluence of 70 to 80%, cells were incubated with UCX® medium containing: i) CM3D; ii) CM2D; and iii) UCX® medium (control), 3× concentrated. Elastin was quantified at 24 hours and 72 hours post-incubation using the Fastin™ Elastin Assay Kit from Biocolor (Carrickfergus, UK), according to the manufacturer’s instructions. The Fastin Assay is a quantitative dye-binding method for the analysis of elastins released into tissue culture medium and extracted from biological materials, namely soluble tropoelastins, lathyrogenic elastins and insoluble elastins (following solubilization to elastin polypeptides α-elastin and κ-elastin). A total of two independent experiments were performed.

#### Gelatin zymography

CM derived from UCX® cultured in either two dimensions (CM2D) or three dimensions (CM3D) and control (10 μg total protein per lane) were separated in a 10% polyacrylamide gel containing 0.1% gelatin as substrate. Precision Plus Protein™ Dual Color Standards (Bio-Rad, Hercules, CA, USA), was used as protein standard. Following electrophoresis, gels were washed twice in 2% Triton X-100 (Sigma-Aldrich) for 30 minutes. After rinsing in H_2_O_dd_, gels were incubated in matrix metalloproteinase (MMP) substrate buffer (50 mM Tris–HCl, pH 7.5; 10 mM CaCl_2_; 0.5% (w/v) NaN_3_) for 16 hours at 37°C. Gels were washed once with H_2_O_dd_ and stained with Coomassie Blue (Sigma-Aldrich) solution for 30 minutes until bands became clear. Band acquisition and density quantification were performed using the Molecular Imager GS800 calibrated densitometer (Bio-Rad). Data was normalized on the protein amount measured in the cell supernatants.

#### Growth factor quantification

The concentrations of hepatocyte growth factor (HGF), fibroblast growth factor (FGF)-2, vascular endothelial growth factor (VEGF)-A, interleukin (IL)-6, TGF-β1, keratinocyte growth factor (KGF) and C-GSF in the CM2D, CM3D and control sample were evaluated by means of a Fluorescent Bead Immunoassay kit (FlowCytomix™, eBioscience) and an enzyme-linked immunosorbent assay (ELISA) kit (Quantikine®, R&D Systems, Minneapolis, MI, USA), for KGF quantification. Protocols were performed as per manufacturer’s recommendations. All samples were acquired on a Gallios (Beckman Coulter) and the results were obtained using FlowCytomix™ Pro 3.0 Software and expressed as pg/mL of total protein, normalized in relation to the control. Results from three independent experiments are shown as fold increase of CM3D relative to CM2D.

#### In vitro scratch assay

HDF and HaCaT cells were seeded into 24-well plates at a seeding density of 1.5 × 10^4^ cells/cm^2^ and 4.5 × 10^4^ cells/cm^2^, respectively, with DMEM with 4 g/L D-(+)-Glucose supplemented with 10% FBS. Once at 80% (for HDF cells) or 60 to 70% confluence (for HaCaT cells), cell media were changed with DMEM with 4 g/L D-(+)-Glucose without FBS for 24 hours. Scratches of ~0.5 mm in width were executed on the monolayer with a sterile pipette tip. Immediately after, the cell surfaces were washed with PBS and maintained in a final volume of 200 μL DMEM with 4 g/L D-(+)-Glucose supplemented either with CM2D, CM3D or control, all 3× concentrated. The area of the scratch, from the same field, was measured at 0 and 40 hours post-scratch as the result of an extensive optimization period of the scratch assay with these two specific cell types. The 40-hour time corresponds to the time period of incubation immediately before the complete scratch closure, and where cells were under the fastest migrating condition. Photographs were taken at an amplification of 40× on a Motic AE2000 inverted microscope. Cellular migration was analysed in the Motic Images Version 2.0 program by calculation of scratch closure, given as the total area occupied by the cells after contact with CM, which was calculated in relation to the initial scratch area at 0 hours. At least nine and six independent experiments in triplicates were performed in HaCaT and HDF, respectively.

#### In vitro tubulogenesis assay

Early passage (P8-P10) human umbilical vein endothelial cells (HUVECs; ScienceCell #8000, Carslbad, CA, USA), were cultured in M199, 1% penicillin (100 mU/mL)/streptomycin (100 μg/mL) solution, supplemented with 10% FBS, 3 μL/mL ECGS (BD Biosciences) and 90 μg/mL heparin. Cells were grown in flasks coated with 0.2% gelatin (Fluka, Buchs, Switzerland), until 70% confluence. The tubulogenesis assay was performed as described in Arnaoutova and colleagues [34] using the thick gel method of preparation. In brief, Matrigel™ growth factor reduced (BD Biosciences) was thawed overnight and poured carefully into eight-well chamberslide LabTeks (Nunc, Wiesbaden, Germany), followed by incubation at 37°C for 45 minutes in order to allow gelification. HUVECs were then inoculated at a density of 4.5 × 10^4^ cells/cm^2^ on top of the Matrigel™ in Endothelial Basal Medium-2 (Lonza Basel, Switzerland), plus 1% penicillin/streptomycin, supplemented (10× concentrated) with basal medium (control) or CM2D or CM3D. Following incubation at 37°C and 5% CO_2_ for 3.5 hours, cells were washed once in PBS and incubated with 3.2 μM Calcein AM (Sigma-Aldrich) for 30 minutes at 37°C. Finally, cells were washed, placed in Endothelial Basal Medium-2 and visualized by fluorescent microscopy using an Axiovert 200 M inverted fluorescence microscope (Carl Zeiss). Tube formation, number of branching points, tube length and thickness were measured using ImageJ (US National Institutes of Health), analysing approximately 25 fields per replicate (n = 3).

#### In vivo wound healing model

Male Wistar rats, 5- to 6-months old, were obtained from Charles River Laboratories. All animals were acclimatized before the experiments and housed in plastic cages under standard laboratory conditions, fed commercial chow and acidified drinking water *ad libitum*.

An excisional wound splinting assay consisting of an adaptation of a protocol already described in mice that was carried out on a rat model [35]. Briefly, after hair removal from the dorsal surface, animals were anaesthetized using intraperitoneal injection of ketamine (75 mg/kg; Imalgene®, Merial, Lyon, France) and medetomidine (0.5 mg/kg; Medetor®, Virbac, Burgdorf, Germany). Full-thickness wounds were performed across the dorsum midline using a sterile 8-mm punch biopsy tool (Kai Medical, Gifu, Japan). To prevent skin contraction, a donut-shaped splint was fashioned from a 0.5-mm thick silicone sheet (Molecular Probes, Carlsbad, CA, USA).

Each animal carried four wounds to which 100 μL of each sample 10× concentrated was applied via subcutaneous injection between the wound margin and the silicone splint of the respective wound, as follows: 1) CM2D; 2) CM3D; 3) control (UCX® medium that was never in contact with cells); and 4) sham (natural wound resolution). Sample administration was repeated after 24 hours, in a total of three applications. Wounds were not covered by any dressing but left to open air. Wound closure was defined as the time at which the wound bed was completely filled with new tissue and re-epithelialized. Wound area was calculated as a percent area of the original wound so that percentage of wound closure was defined as follows: (area of original wound – area of actual wound)/area of original wound × 100, being the wound area measured by tracing the wound margin and calculated using an image analysis program (ImageJ). Figures show representative images of three independent experiments using five to six animals per time point.

### Wound histological analysis

Animals were sacrificed at days 7, 9 and 14 for histological analysis. The wound area was excised and fixed in 10% neutral buffered formalin (Sigma-Aldrich) for haematoxylin and eosin (H&E) staining. As part of the histological evaluation, all slides were blindly examined by a pathologist. A histological score was given to each slide according to the parameters summarized in Table 1 (re-epithelialization, granulation tissue formation, vascularization and wound margins distance).

**Table 1:** **Criteria for histologic scoring of wound healing**

**Score**	**Re-epithelialization**	**Wound margins distance**	**Granulation tissue**	**Vascularization**
0	<25% of re-epithelialization	Distant wound margins	Absent granulation	Presence of haemorrhage
1	25-50% of re-epithelialization	Distant wound margins by granulation tissue	<30% of granulation tissue	Presence of haemorrhage and capillaries
2	50-75% of re-epithelialization	Moderate distance between wound margins	Granulation in 30-60% of wound bed	Presence of many capillaries
3	>75% of re-epithelialization	Reduced distance between wound margins	>60% of granulation tissue	Presence of few capillaries
4	Complete re-epithelialization	Closed wound margins	Traces of granulation with presence of mature collagen fibres	No evident vascularization

### Statistical analysis

Data analysis and graphs were plotted using GraphPad Prism® software (GraphPad Software, San Diego, CA, USA). Values presented in the text and figures are as mean ± standard deviations of at least three independent experiments, except otherwise specified. To estimate the significance of the differences of the growth factor quantification and of the data obtained in the *in vivo* assay, the parametric students *t* test was used. The one way analysis of variance test using Tukey’s *post-hoc* test for correction of multiple comparisons was also performed. *P* < 0.05 was considered significant.

## Results

### UCX® form viable spheroids in spinner flask suspension culture

**Figure 1:**
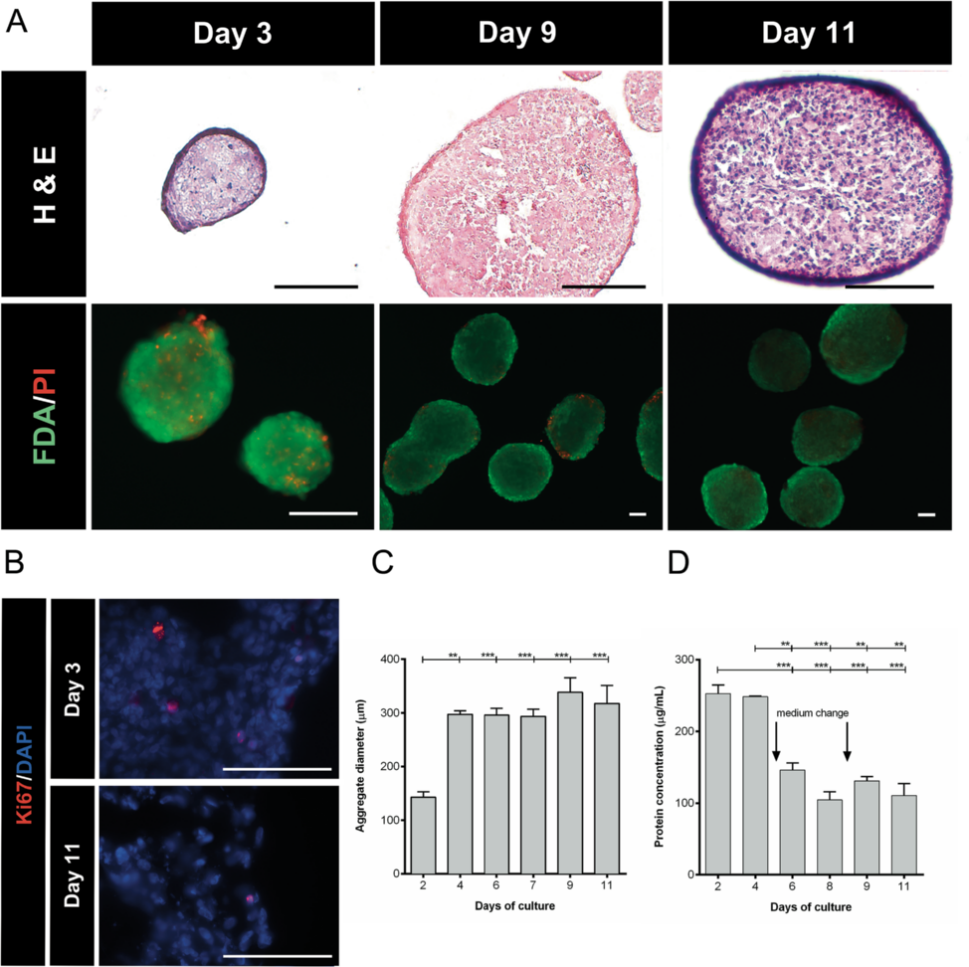
Spinner flask suspension cultures allow for the extended maintenance of UCX® spheroids without necrotic centres. **(A)** Phase contrast and fluorescence representative images of spheroids at days 3, 9 and 11. (Top) Haematoxylin and eosin (H&E) staining of UCX® spheroid sections. Scale bar = 100 μm; n = 3. (Bottom) Viability of UCX® spheroids in culture as assessed by staining with fluoresceine diacetate (FDA; live cells, green) and propidium iodide (PI; dead cells, red). Scale bar = 100 μm; n = 3. **(B)** Representative immunofluorescence images of UCX® spheroid cryosections labelled with Ki-67 (red) at days 3 and 11 in culture. Nuclei were labelled with DAPI (blue). Scale bar = 100 μm; n = 3. **(C)** Sizes of UCX® spheroids at days 2, 4, 6, 7, 9 and 11. Sizes were measured from 7 to 13 captured images of spheroids. Spheroids reached an average size of 308 ± 9.84 μm from day 4 onwards. Data are shown as mean ± standard error of the mean; n = 3. **(D)** Biomass quantification measured by BCA kit at days 2, 4, 6, 8, 9 and 11. Data are shown as mean ± standard deviation; n = 3. **P < 0.01; ***P < 0.001.

A SFSC system was developed and optimized in order to obtain UCX® three-dimensional spheroids (Figure 1). On average, spheroid diameters were 143 ± 9.78 μm (value ± standard error of the mean (SEM)) after 2 days and 309.5 ± 9.38 μm (value ± SEM) from day 4 to day 11 of culture (Figure 1C). H&E staining revealed compact spheroids with cells evenly distributed and embedded in ECM presenting absence of an inner necrotic core up to day 11, indicating that cells are viable within the core of spheroids (Figure 1A). The surface of the spheroid had a layer of epithelium-like cells that were flatter and more elongated in appearance. Ki67 staining of spheroid cryosections showed the presence of proliferating cells within spheroids at days 3 and 11 (Figure 1B). However, Ki67-positive cells comprised only a small fraction of cells, indicating that only a low fraction (<5%) of cells were actively proliferating in spheroids and this fraction of proliferating cells decreases with time (Figure 1B). The observed residual cell proliferation is in agreement with the biomass values that did not significantly change during culture time. The exception was between days 4 and 6 when the biomass value decreased due to medium change that inevitably resulted in loss of still non-aggregated cells (Figure 1D). From day 6 onwards, where no single cells could be observed, no significant differences were detected in biomass quantification and no loss of biomass was detected after medium change at day 9. The results showed that our optimized culture conditions enabled the formation and maintenance of UCX® spheroids comprising viable cells for at least 11 days and during the period of medium conditioning.

### UCX® grown in three-dimensional culture conditions maintain mesenchymal stromal cell antigen expression phenotype

UCX® cell-surface marker expression was analysed by flow cytometry (see Additional file 1: Figure S1A). The surface epitopes of UCX® dissociated from spheroids were similar to the surface epitopes of UCX® obtained from adherent monolayers (two-dimensional) dissociated under the same conditions. From day 6 onwards, the population of three-dimensional spheroid-dissociated cells showed a decrease in CD105 and CD90 expression levels. In fact, flow cytometry side scatter results indicate that cells grown in three-dimensional spheroids were approximately 30% smaller in size when compared to cells grown in two-dimensional monolayer cultures (results not shown). The expression of CD105 and CD90 surface epitopes increased again to high levels once UCX® grown in three-dimensional spheroids were plated back (from culture day 7) in monolayer conditions (spheroids plated back in two-dimensions; see Additional file 1: Figure S1A).

### UCX® cultured as spheroids maintain mesenchymal stromal cell differentiation potential after being plated back in two-dimensional culture conditions

In order to assess if three-dimensional culture conditions altered hallmark properties of UCX®, namely their differentiation potential, cells in three-dimensional culture were dissociated from spheroids at days 3, 6 and 9, plated onto culture flasks and grown as monolayers. Plated cells retained the ability to adhere and proliferate on a plastic surface. Cell multipotency was then assessed and confirmed by the ability of UCX® to differentiate *in vitro* into adipocyte-, osteoblast- and chondrocyte-like cells (see Additional file 1: Figure S1B). Adipogenic and osteogenic biochemical differentiation, evidenced by lipid vacuole formation and matrix mineralization, respectively, could be confirmed at all time points. In turn, chondrogenic differentiation was attempted using both three-dimensional spheroid-dissociated cells and intact three-dimensional spheroids directly. As expected, chondrocyte differentiation was obtained with dissociated cells, but was greatly enhanced by cells in aggregates already embedded in their own chondrogenic-type ECM. Overall, cells obtained from three-dimensional cultures and plated back under two-dimensional conditions show a similar differentiation capacity as cells grown in conventional two-dimensional cultures at similar passages.

### UCX® spheroid structures mimic the native environment *ex vivo*

**Figure 2:**
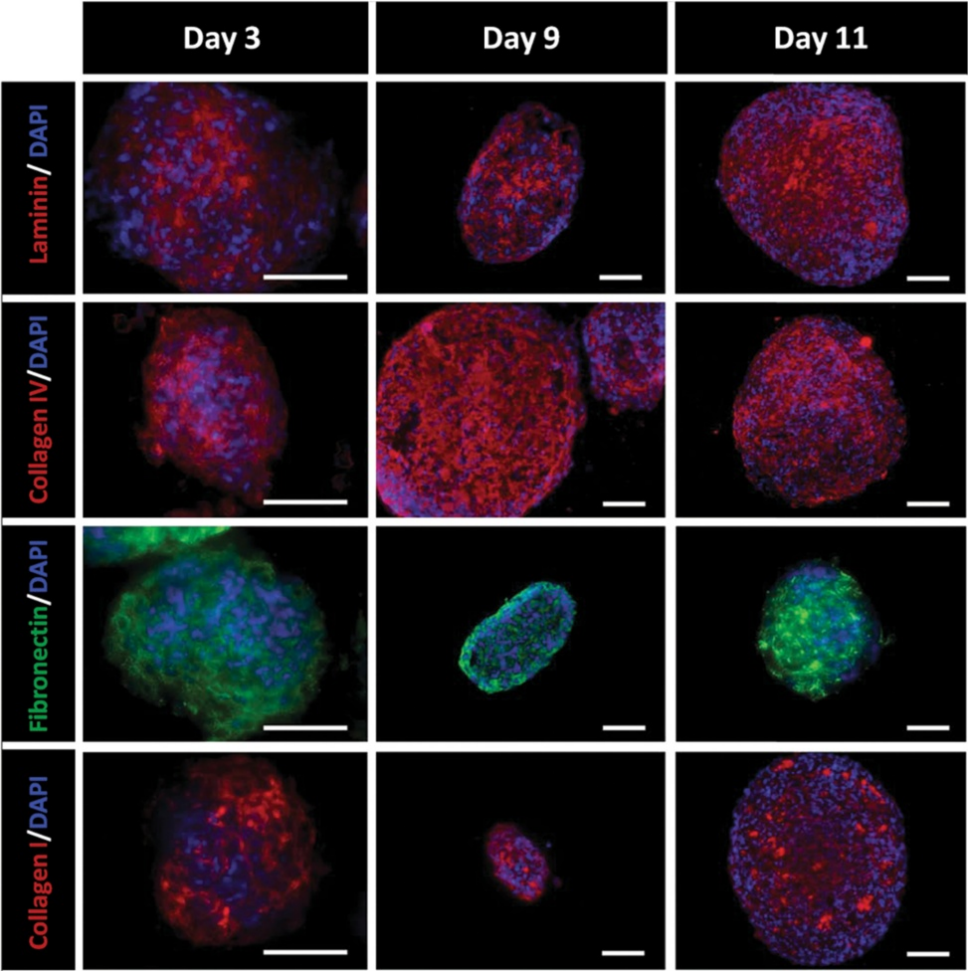
Expression of extracellular matrix proteins by UCX® spheroids. Immunostaining of representative cryosections of UCX® grown in three-dimensional culture demonstrate the expression of relevant extracellular matrix (ECM) molecules. Within the spheroid, laminin and collagen IV define the basal lamina surrounding UCX® which is in close association with the ECM proteins fibronectin and collagen I. A similar ECM composition was observed irrespectively of the culture duration when considering the analysed time-points of day 3, 9 and 11. Scale bar - 100 μm; n = 3.

To evaluate whether the UCX® spheroids replicate the native tissue environment *ex vivo* by producing the Wharton’s jelly ECM, the expression of major ECM proteins and the activity of MMP was analysed. Immunohistochemical evaluation of cells in spheroids showed sustained expression of the ECM components collagen I and fibronectin, as well as of the basement membrane proteins laminin and collagen IV, between days 3 and 11 *in vitro* (Figure 2). Moreover, no differences in terms of ECM distribution/composition between the aggregate inner core and outer layer in any of the different-sized aggregates were observed. A uniform distribution of laminin, collagen IV and fibronectin expression, with interspersed regions of collagen I deposition, generically characterized the UCX® spheroids.

**Figure 3:**
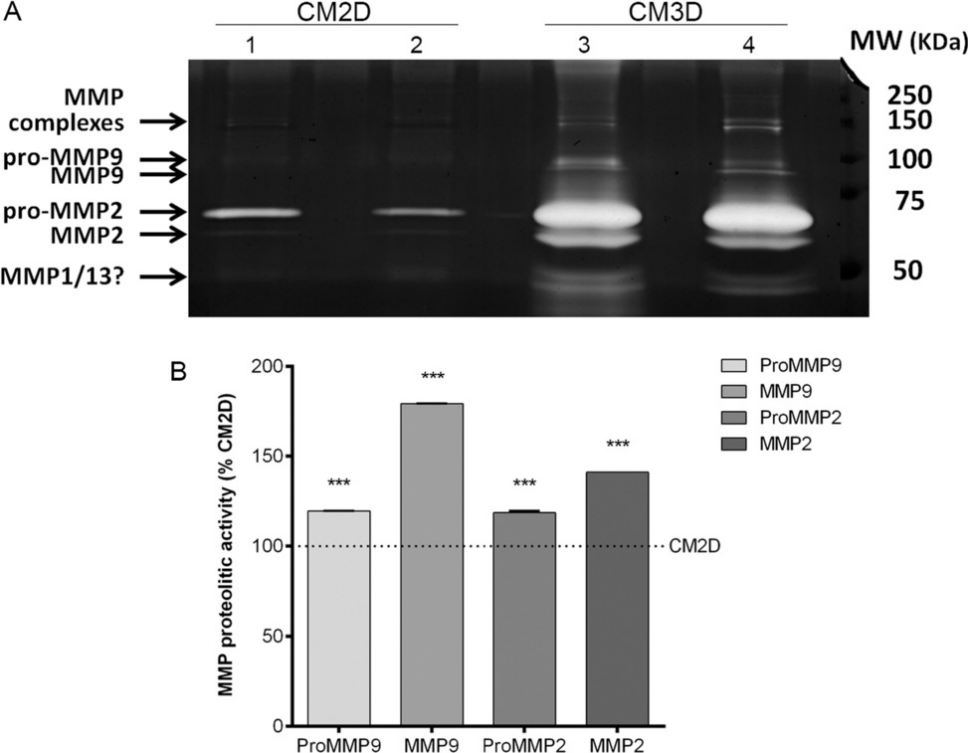
The production and secretion of matrix metalloproteinases (MMPs) was increased in UCX® cells within spheroids. Gelatin zymography of conditioned medium produced by UCX® cells in two-dimensional (CM2D; lanes 1 and 2) or three-dimensional (CM3D; lanes 3 and 4) culture conditions. **(A)** Zymogram analysis demonstrates that different culture conditions result in variations of the content of produced MMPs. All the samples display a high molecular weight (~130 kDa) set of bands depicting MMP complexes, and also single MMPs such as pro-MMP-9 (~92 kDa), MMP-9 (~82 kDa), pro-MMP-2 (~72 kDa), MMP-2 (~66 kDa) and an additional low molecular weight (~45 kDa) set of bands that could refer to other MMPs with residual proteolytic activity in gelatin substrates (possibly MMP-1 or −13). **(B)** Quantification of proteolytic bands by densitometric analysis of three independent experiments (n = 3) indicates that UCX® seeded under three-dimensional conditions produce higher relative amounts of MMP-9 and MMP-2, both the zymogen and the active isoform. ***P < 0.001.

Gelatin zymography analysis demonstrated that UCX® spheroids secreted the latent zymogen as well as the respective active enzyme of MMP-2 (72 kDa and 66 kDa, respectively) and MMP-9 (92 kDa and 82 kDa, respectively) (Figure 3A). When compared to the CM obtained from adherent cultures (CM2D), higher activity of MMP-2 and MMP-9 forms was detected on CM3D (1.41-fold and 1.79-fold, respectively). Moreover, densitometry analysis of the proteolytic bands further confirmed significant differences in the amount of the zymogen and active MMP-2 and MMP-9 isoforms between the two CM in three independent experiments (Figure 3B). The presence of other gelatinolytic MMPs was detected in CM3D, and in very low amounts in the CM2D, with a molecular weight of ~45 kDa which is compatible with either MMP-1 activity or MMP-13 residual gelatinase activity (Figure 3A).

### UCX® spheroids present a secretome richer in trophic factors involved in wound healing

**Figure 4:**
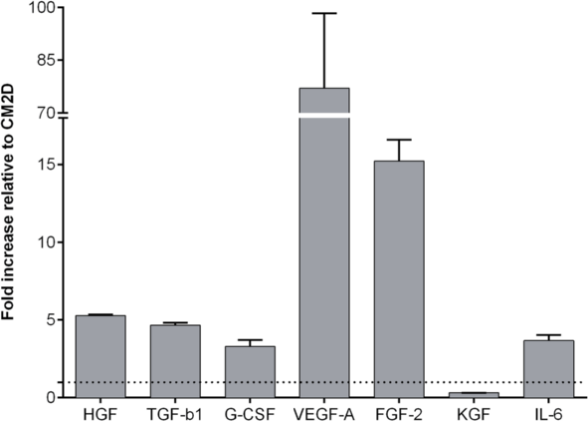
The secretion of growth factors involved in the wound-healing process was increased in UCX® spheroids. Hepatocyte growth factor (HGF), transforming growth factor (TGF)-β1, granulocyte-colony stimulating factor (G-CSF), vascular endothelial growth factor (VEGF)-A, fibroblast growth factor (FGF)-2 and interleukin (IL)-6 were quantified in two-dimensional conditioned medium (CM2D) and three-dimensional conditioned medium (CM3D) using a FlowCytomix™ kit. Keratinocyte growth factor (KGF) was quantified in CM2D and CM3D by enzyme-linked immunosorbent assay. Results are expressed as fold-increase of CM3D relative to CM2D. Data are shown in mean ± standard deviation; n = 3.

In order to evaluate whether our three-dimensional culture conditions direct UCX® to secrete a trophic factor profile richer in wound healing-related factors, when compared to two-dimensional cultures, a comparative analysis of seven representative trophic factors with vital importance in the consecutive stages of wound healing was performed. VEGF-A, TGF-β1, KGF, FGF-2, IL-6, G-CSF and HGF were quantified from CM3D and CM2D produced in similar conditions in terms of relative amount of cells, having undergone an equivalent number of cPDs, conditioning volume and conditioning time, and eliminating serum contribution. UCX® grown and maintained either in spheroids or monolayers resulted in different secretome profiles. In particular, the levels of HGF, TGF-β1, IL-6 and G-CSF found in CM3D were higher than in CM2D (Figure 4). Finally, a 15-fold increase in FGF-2 levels was observed in CM3D when compared to CM2D. Most impressively, VEGF-A, which was only residually expressed in the two-dimensional system, was highly expressed by UCX® under three-dimensional conditions (80-fold higher than CM2D). In turn, KGF expression was found to be significantly higher in CM2D versus CM3D. Overall, the results strongly suggested an improved paracrine effect of CM3D onto fibroblast-mediated ECM synthesis, angiogenesis and vasculogenesis, essential for the granulation tissue formation and remodelling stages of wound healing [36].

### The UCX® paracrine induction of skin cell migration, proliferation and tubulogenesis *in vitro* is enhanced when cells are cultured as spheroids

**Figure 5:**
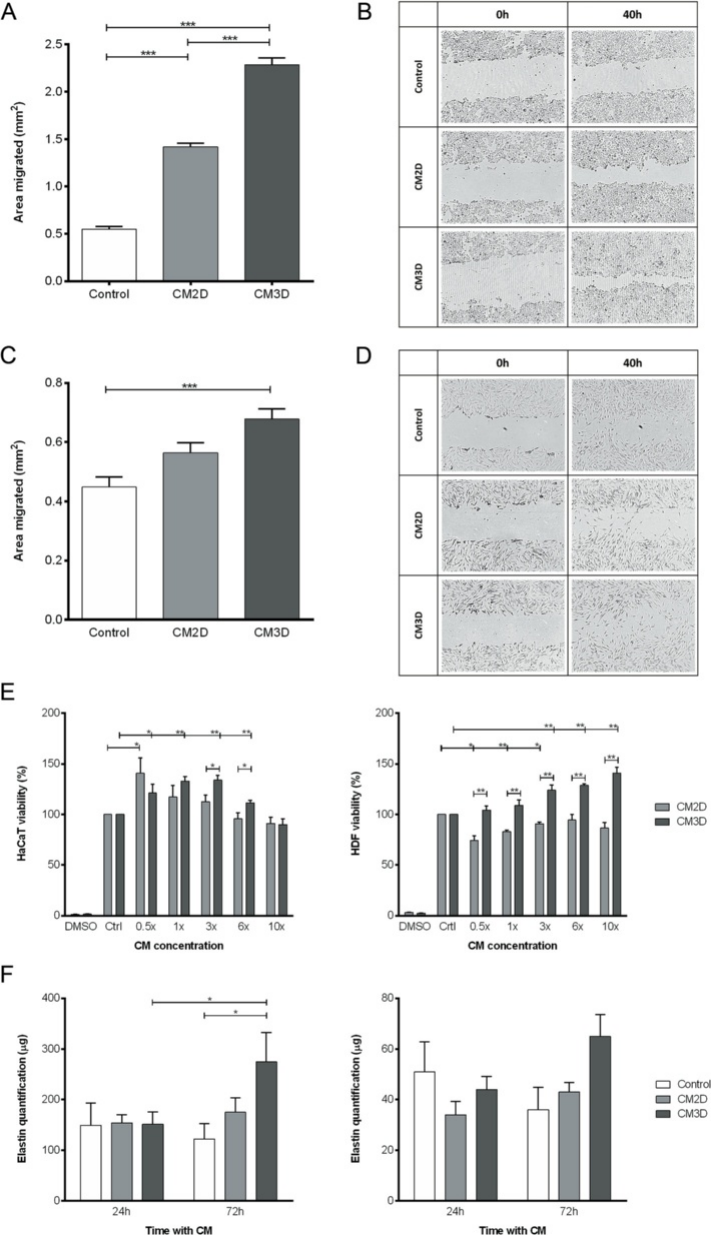
UCX® spheroid-derived conditioned medium (CM3D) enhances immortalized keratinocyte cell line (HaCaT) and human dermal fibroblasts (HDF) cell migration, proliferation and elastin production. **(A)** Graphs represent the average area occupied by the HaCaT cells after 40 hours in contact with serum-free conditioned medium from three-dimensional cultures (CM3D), two-dimensional cultures (CM2D) and control (basal medium). Data are shown as mean ± standard deviation (SD); n = 9 to 12. **(B)** Representative images of HaCaT scratch-wound assays immediately after the scratches had been made (0 h) and then after 40 hours in the presence of serum-free CM3D, CM2D and control. **(C)** Graphs represent the average area occupied by the HDF after 40 hours with CM3D, CM2D and control. Data are shown as mean ± SD; n = 6 to 9. **(D)** Representative images of HDF scratch-wound assays immediately after the scratches had been made (0 h) and then after 40 hours in the presence of serum-free CM3D, CM2D and control. **(E)** HaCaT and HDF viability evaluated by methylthiazolyldiphenyl-tetrazolium bromide (MTT) reduction assay after 48 hours of incubation with CM2D and CM3D. Data are shown as percentage of control (Crtl) and as mean ± standard error of the mean; n = 3. **(F)** Elastin production by HaCaT and HDF at 24 and 72 hours post-incubation with CM3D, CM2D and control. Data are shown as mean ± SD; n = 2. *P < 0.05; **P < 0.01; *** *P* < 0.001. CM, conditioned medium.

**Figure 6:**
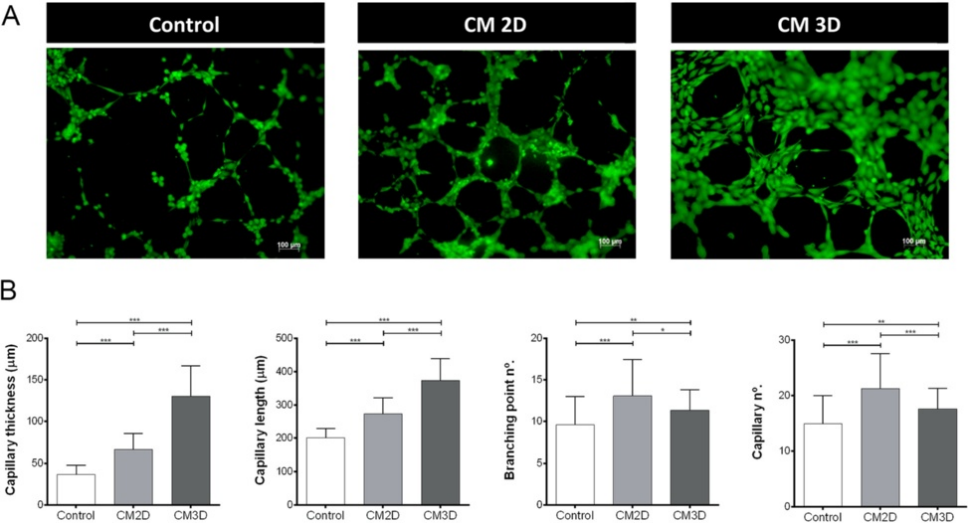
UCX® spheroid-derived conditioned medium (CM3D) enhances capillary formation *in vitro*. **(A)** Representative images of Calcein AM marked human umbilical vein endothelial cells on Matrigel, in contact with control (basal media), CM2D and CM3D. **(B)** Graphs represent a quantitative analysis of capillary thickness, length, branching point number and capillary number. Data are shown as mean ± standard deviation; n = 3. *P < 0.05; **P < 0.01; ***P < 0.001. CM2D, two-dimensional conditioned medium; CM3D, three-dimensional conditioned medium.

The functional effects of CM produced by UCX® cultured as spheroids were investigated regarding three essential aspects of skin regeneration; that is, skin cell migration, proliferation and angiogenesis. The capacities for cellular migration and proliferation of keratinocytes (by means of the HaCaT cell line) and dermal fibroblasts (HDF) were quantified using *in vitro* scratch and cell viability assays, respectively. Moreover, the sprouting of new capillaries was assessed by the formation of capillary-like structures by endothelial cells (HUVECs) using classical Matrigel™ assays. The keratinocyte migration into the scratch wound area was accelerated in the presence of CM3D when compared to CM2D (Figure 5A). Using digital image analysis, we have quantified the scratch areas that have been closed resulting in a statistically significant (*P* < 0.001) increase in HaCaT migration, in the presence of CM3D, of approximately two- and four-fold when compared to CM2D and control samples, respectively (Figure 5A,B). In addition, CM3D significantly induced HaCaT proliferation (at all concentrations except 10×, *P* < 0.01) while CM2D resulted only in a slight mitogenic effect (*P* < 0.05 for 0.5× concentration; Figure 5E). With regards to the migration of HDF, although not statistically significant, CM3D also provided a noticeable increase in cell migration when compared to CM2D (Figure 5C,D). In turn, according to our cell viability assays, HDF viability was enhanced by CM3D up to 40% compared to the control, whereas CM2D revealed no considerable effect on HDF viability, especially at higher concentrations (6× and 10×; Figure 5E). Furthermore, consistent with our scratch assay results, CM3D was found to enhance the production of elastin in both HaCaT and HDF (Figure 5F), a protein known to be involved in multiple functions during wound healing by virtue of its mechanical and signalling properties. After 72 hours in culture, the increase in elastin concentration was statistically significant in HaCaT cells both with respect to elastin concentration at 24 hours in culture and with respect to the control at 72 hours. We have further addressed whether CM3D would induce the *in vitro* formation of capillary-like structures by endothelial cells (HUVECs). Tubule formation by HUVECs consistently showed a significant increase in capillary number, thickness and length, and number of branching points in the presence of CM3D when compared with control (*P* < 0.001; Figure 6A,B). Interestingly, while CM3D-supplemented medium still resulted in significantly thicker and longer capillary structures when compared to CM2D, the latter slightly induced the formation of more capillaries and branching points (Figure 6A,B). Overall, these results demonstrate for the first time that UCX® favour maturation of neo-formed capillaries via a paracrine mechanism, and that this activity is further increased by culturing UCX® as spheroids in our optimized three-dimensional setting.

### Three-dimensional conditioned medium enhances the wound-healing capacity *in vivo* in a rat excisional wound splinting model

**Figure 7:**
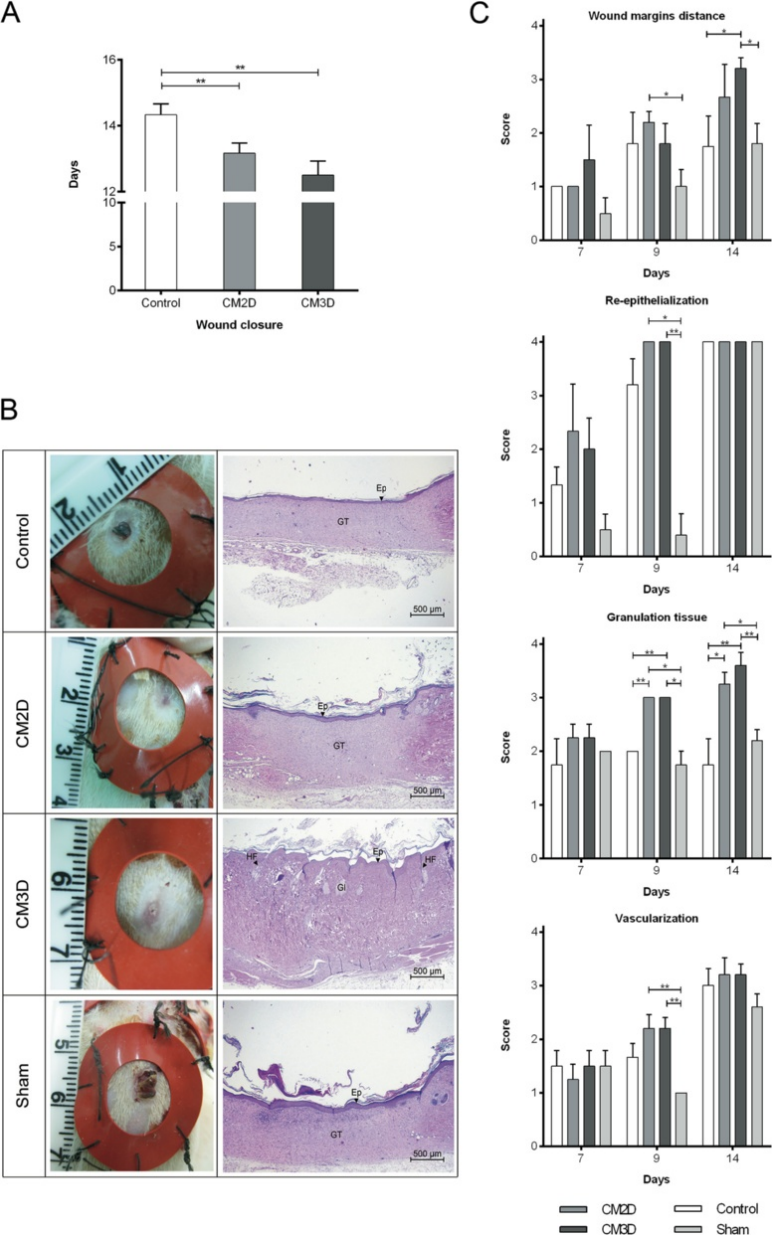
UCX® three-dimensional culture-derived conditioned medium (CM3D) enhanced wound healing *in vivo*. **(A)** Graph represents the day of closure of wounds treated with two-dimensional conditioned medium (CM2D), CM3D and control. Data are shown as mean ± standard error of the mean (SEM); n = 6. **(B)** Representative images (left) of macroscopic view and (right) histological slices stained by haematoxylin and eosin of CM2D-treated, CM3D-treated, control and sham-treated wounds at day 14; n = 6. **(C)** Graphs represent the histological scores (top to bottom) of wound margin distance, re-epithelialization, granulation tissue and vascularization, evaluated in CM2D-, CM3D-, control and sham-treated wounds at days 7 (n = 4), 9 (n = 5) and 14 (n = 6). Data are shown as mean ± SEM. *P < 0.05; **P < 0.01. GT, granulation tissue; Ep, epithelium; HF, hair follicle; Gl, gland.

To examine whether CM could enhance wound healing *in vivo* consistent with the potential therapeutic effects observed *in vitro*, an excisional wound splinting assay was carried out using Wistar rats as an animal model. Macroscopic observations showed that CM-treated wounds clearly exhibited accelerated wound closure when compared to both types of control wounds. Full closure in wounds treated with CM3D and CM2D was significantly faster than the control, having been reached at days 12 and 13, respectively (Figure 7A). Additionally, a thorough histological analysis resulted in the observation of significant differences between CM-treated and CM-untreated wounds, especially with regards to granulation tissue formation from day 9 onwards (Figure 7B,C). Furthermore, at day 9, CM2D- and CM3D-treated wounds already presented signs of faster wound resolution, given the higher scores observed for wound margin distance (*P* < 0.05 for CM3D), complete re-epithelialization (*P* < 0.05 and *P* < 0.01, respectively) and higher vascularization level (*P* < 0.01 for both CM2D and CM3D), when compared to the natural resolution (sham) control. Indeed, both controls could only achieve a highest histological score for the re-epithelialization parameter and only at day 14 (Figure 7C). In turn, the evaluation of other parameters related to new tissue formation have revealed slightly different healing stages between CM3D- and CM2D-treated wounds. In general, CM3D-treated wounds at day 14 presented a tissue which appeared completely regenerated, with a more mature vascular system, based on the presence of several organized capillaries, and already showing cell appendages such as glands and hair follicles (Figure 7B). Meanwhile, CM2D-treated wounds still presented on-forming granulation tissue with some mature collagen fibres being observed and both controls still on the verge of full closure (Figure 7B). Overall, the results demonstrate that CM from UCX® cultured in our three-dimensional conditions promotes an acceleration and improvement of the wound-healing process.

## Discussion

Increasing evidence supports that MSCs have a role in tissue repair and regeneration, not so much by means of cellular differentiation but rather via the secretion of soluble trophic factors that enhance the response of damaged tissues through paracrine activation of surrounding cells [8,37,38]. Most of the MSC application studies performed so far have been performed using MSCs cultured in static monolayer cultures. However, several publications suggest that a three-dimensional environment may be more physiologically relevant than traditional two-dimensional systems mainly by increasing cell-to-cell interactions. Indeed, previous work has shown that WJ-MSCs have higher wound-healing capabilities when seeded on membrane scaffolds prior to transplantation onto skin injuries *in vivo*, demonstrating that a three-dimensional environment can prime WJ-MSCs to a more therapy-driven phenotype [24,39]. However, the use of scaffolds or other attachment materials often adds on regulatory hurdles to the already intrinsically complex process of approving advanced therapy medicinal products. Alternatively, MSCs have been cultured and maintained in self-assembled spheroids. Such cellular structures have demonstrated improved therapeutic potential by increasing the expression of C-X-C chemokine receptor type 4, IL-24 or tumour necrosis factor-inducible gene 6 protein and prostaglandin E_2_ genes that promote adhesion to endothelial cells, and have tumour suppressing or anti-inflammatory properties, respectively [1,19,40,41]. Along this line of thought, the work presented here aimed at: i) establishing a consistent protocol for obtaining UCX® spheroids with an improved “healing” secretome compared to that obtained from UCX® expanded and maintained in traditional monolayer cultures; and ii) to use the medium conditioned by such UCX® spheroids as the therapeutic agent for the treatment of open skin wounds.

UCX® spheroids were successfully cultured and maintained in suspension using a spinner flask with a ball impeller. The formation of necrotic centres that may occur in aggregates larger than 350 μm was a concern [42]. This was circumvented by controlling the stirring rate, as shown in the H&E analysis. In fact, under our optimized conditions, spheroids maintained high cell viability for up to at least 11 days, whereas cell proliferation was stalled (<5%) when compared to monolayer cultures. Additionally, the MSC character of the UCX® in spheroids promoted stemness maintenance as per the recommendations of the International Society for Cellular Therapy [14]. A decrease in CD105 and CD90 expression levels was the exception, most likely due to the smaller cell size while in spheroids which, by resulting in a lower yield of fluorescence emission, shifted the signal to the negative region of the histograms - an observation previously reported by Bartosh and colleagues [1]. The dynamics of cell aggregation are mediated by cell adhesion molecules, where single cells firstly aggregate through integrin binding into loose aggregate spheroids. Afterwards, it has been shown that E-cadherin interactions are the major factor for the cell morphological transition from loose cell aggregates to compact spheroids. Therefore, cells within spheroids may be smaller in size than cells grown in monolayer cultures [43,44].

Moreover, evidence indicates that a crucial component of the stem/progenitor cell niche is the ECM which modulates cell fate by making available a large array of biochemical and biophysical cues. Wharton’s jelly is a connective tissue layer very rich in ECM fibrous proteins, interstitial proteins and signalling molecules, whereas the WJ-ECM is mainly composed of collagens such as collagen type I, II, III, IV and VI, laminin and fibronectin [45]. Immunostaining of UCX® spheroids showed that cells were embedded in a rich ECM composed of mainly collagen I, fibronectin, laminin and collagen IV, suggesting that an environment resembling that of the native tissue was formed within the aggregates. Besides, it has been demonstrated that the preservation of an endogenous ECM in human MSC aggregates improves multi-lineage potential and survival under ischaemic stress [45]. Thus, the production of ECM within the aggregate may have also increased the overall cell homeostasis, preserving cells from entering apoptosis.

Previous studies using mRNA/cDNA microarrays demonstrated marked differences between transcriptome profiles of MSCs maintained in spheroids versus MSCs cultured in monolayers, including those confirming widespread changes to the cellular architecture and ECM [19,40,46]. The fact that the cell proliferation/metabolism is rather different between the two systems may also have an important effect upon the overall cellular activity leading to large differences in the secretion of relevant paracrine factors. Herein, a representative group of growth factors was selected, involved in important mechanisms of tissue regeneration, including all wound-healing phases from tissue haemostasis to remodelling. The corresponding analysis was performed in samples concentrated 10× using a 3-kDa cut-off; that is, lower than the molecular weight of the selected factors. According to our quantification analysis, VEGF-A, FGF-2, HGF, TGF-β1, G-CSF and IL-6 are consistently secreted in higher amounts by UCX® maintained in spheroids, when compared to UCX® grown in static monolayer cultures. In turn, and according to our previous results where CM2D was found to have a prominent motogenic effect on keratinocytes and therefore on the early epithelization stage of wound healing [12], KGF expression was significantly higher in CM2D when compared to CM3D. Of special note was the high production by UCX® spheroids of VEGF-A and FGF-2, both conspicuous growth factors involved in neo-vascularization and regulation of tissue homeostasis, respectively [47]. Expression of both VEGF-A and FGF-2 was only residually detected in UCX® grown and maintained in two-dimensions in our experimental conditions. This is consistent with previous findings by Potapova and colleagues [46] using hanging drop human MSCs three-dimensional aggregates [46]. In turn, HGF and TGF-β1 have also been highly induced by our three-dimensional culture conditions. Both factors have been implicated in keratinocyte cell migration across the fibronectin-rich provisional wound matrix during the healing process by promoting MMP, ECM protein, integrin and collagenase secretion [48-53]. TGF-β1 has also diverse stimulatory roles in cutaneous wound healing, namely in re-epithelialization, wound contracture, vasculogenesis, scar formation and ECM deposition [7]. Many of these processes involve the regulation of elastin expression which plays a vital role in tissue structure and function by inducing a range of cell activities, including cell migration and proliferation, matrix synthesis, and protease production. When elastin is inadequately expressed due to wound healing, an intact elastic fibre network is absent contributing to poor remodelling and therefore diminished physical properties when compared with uninjured skin. After injury, the restoration of an intact and functional elastic fibre network is therefore critical to regain complete skin function [54]. Herein, CM3D induced elastin production by HaCaT and HDF over time, suggesting a UCX®-CM contribution to the synthesis of a functional elastin fibre network. Furthermore, improvement in MMP-9 activity, together with augmented HGF secretion, stimulated by our three-dimensional culture conditions, may also be at the basis of the CM3D-mediated keratinocyte/fibroblast migration observed in our *in vitro* migration and proliferation assays. MMP-9 has been implicated in enhanced invasive potential of keratinocytes in response to EGF and HGF [55]. The secretion of both MMP-2 and MMP-9 has been associated with MSC recruitment and infiltration into injured tissues *in vivo* [56]. Thus, the higher production of both MMP-9 and MMP-2 by UCX® spheroids may reflect the requirement to surmount the complex ECM mesh formed within the spheroid when compared to the plane basement membrane formed in monolayers.

Correct cutaneous wound repair requires a well-coordinated response of platelet recruitment, inflammation (infiltrate-cell mobilization), cell migration and granulation tissue formation, neovascularization, ECM degradation/formation, and epithelialization. Failure of any of these processes due to ischaemia, reperfusion injury, bacterial infection, or ageing can result in chronic inflammation and/or a non-healing wound [6,57]. Previously we have observed an important role of CM2D produced by UCX® in the early epithelialization stages of cutaneous wound healing due to the expression of G-CSF, endothelial growth factor, FGF-2 and KGF, and their impact in the induction of keratinocyte activity [12]. The role of CM2D produced by UCX® on the later proliferation and remodelling stages of wound healing seemed to be more indirect, by recruiting other local and circulating MSCs (such as BM-MSCs) via a G-CSF-mediated mechanism [12]. Herein, the CM3D-mediated induction of VEGF-A and FGF-2, together with increased expression of HGF and TGF-β1, strongly supports a CM3D-specific enhancement of fibroblast proliferation and function, with concomitant enhancement of the granulation process. Also, the high expression levels of VEGF-A obtained in CM3D (non-detected in CM2D in our experimental conditions) indeed suggested an increased potential to induce angiogenesis and vasculogenesis *in vivo*, corroborated by our *in vitro* tubulogenesis results [58]. Additionally, G-CSF, with important implications in platelet and infiltrate cell recruitment, keratinocyte migration and function as well as mobilization of resident and circulating haematopoietic and MSCs [7], could also be detected at higher levels in CM3D. In fact, both CM3D- and CM2D-treated wounds *in vivo* presented accelerated closure (around 17% and 14%, respectively) showing complete re-epithelialization and higher vascularization levels when compared to the controls. However, unlike CM2D-treated wounds, CM3D-treated post-closure wounds, 14 days after excisional infliction, presented a completely regenerated tissue, with a mature vascular system with organized capillaries, but already showing glands and hair follicles. Taken together, the CM3D biochemical composition seems to have promoted the later proliferative and remodelling stages of wound healing, when compared to CM2D, which can explain the advanced tissue regeneration profile observed *in vivo*.

## Conclusions

A reproducible and scalable SFSC system for the maintenance of viable, multipotent UCX® within self-assembled spheroids was developed. The microenvironment established within the spheroids acted in an autocrine fashion favouring an enhanced secretion of healing-inducing paracrine factors by UCX®, including the ECM proteins, collagen I, fibronectin, laminin and collagen IV; MMP-2, MMP-9, VEGF-A, G-CSF, HGF, FGF-2, TGF-β1 and IL-6. The resulting conditioned medium (CM3D) has the potential to increase elastin production, skin cell mobility and further induce tubulogenesis *in vitro*. The enhanced CM3D healing potential *in vitro* was corroborated *in vivo* in an excisional wound splinting model, resulting in a faster wound closure and regeneration into a more mature fully functional tissue. Finally, by resorting to a reproducible and scalable three-dimensional culture system, this work presents an important advantage with regards to an eventual CM production for clinical and industrial applications.

## Additional file

Additional file 1: Figure S1. Figure presenting the data regarding the surface protein expression and tri-lineage differentiation of UCX® spheroids, as described in the corresponding figure legend: UCX® spheroids retain the properties of MSCs from adherent cultures. **(A)** Flow cytometry of surface protein expression on UCX® spheroids at days 3 (D3), 6 (D6), 9 (D9) and 11 (D11) in culture; on UCX® cells collected from spheroids from three-dimensional cultures at day 7 and plated back into two-dimensional cultures; and on UCX® originally cultured in two-dimensional conditions. Cells were dissociated from spheroids with Trypsin/EDTA prior to flow cytometry analysis; n = 4. **(B)** Representative images of the differentiation of UCX® spheroids (three-dimensional) into osteoblast-like cells, chondrocyte-like cells, adipocyte-like cells and respective controls (two-dimensional). Cultures were stained with alkaline phosphatase, alcian blue and oil red O, respectively. Scale bar = 100 and 400 μm.

## Abbreviations

BM-MSC: bone marrow-derived mesenchymal stromal cell
BSA: bovine serum albumin
CM: conditioned medium
CM2D: two-dimensional conditioned medium
CM3D: three-dimensional conditioned medium
cPD: cumulative population doubling
DMEM: Dulbecco’s modified Eagle’s medium
DMSO: dimethyl sulphoxide
ECM: extracellular matrix
EDTA: ethylenediamine tetraacetic acid
FBS: foetal bovine serum
FGF: fibroblast growth factor
FITC: fluorescein isothiocyanate
G-CSF: granulocyte-colony stimulating factor
H&E: haematoxylin and eosin
HaCaT: immortalized keratinocyte cell line
HDF: human dermal fibroblasts
HGF: hepatocyte growth factor
HUVEC: human umbilical vein endothelial cell
IL: interleukin
KGF: keratinocyte growth factor
MEM: modified Eagle’s medium
MMP: matrix metalloproteinase
MSC: mesenchymal stromal cell
MTT: methylthiazolyldiphenyl-tetrazolium bromide
P: passage
PBS: phosphate-buffered saline
PE: phycoerythrin
SEM: standard error of the mean
SFSC: spinner flask suspension culture
TGF: transforming growth factor
VEGF: vascular endothelial growth factor
WJ-MSC: Wharton’s jelly-derived mesenchymal stromal cell
