# Supplementary material for: Three-dimensional spheroid cell culture of umbilical cord tissue-derived mesenchymal stromal cells leads to enhanced paracrine induction of wound healing
Source: Stem Cell Res Ther. 2015 May 9;6(1):90. doi: 10.1186/s13287-015-0082-5 (PMC4448539; doi:10.1186/s13287-015-0082-5)
Supplement: Additional file 1: Figure S1. — Figure presenting the data regarding the surface protein expression and tri-lineage differentiation of UCX® spheroids, as described in the corresponding figure legend: UCX® spheroids retain the properties of MSCs from adherent cultures. (A) Flow cytometry of surface protein expression on UCX® spheroids at days 3 (D3), 6 (D6), 9 (D9) and 11 (D11) in culture; on UCX® cells collected from spheroids from three-dimensional cultures at day 7 and plated back into two-dimensional cultures; and on UCX® originally cultured in two-dimensional conditions. Cells were dissociated from spheroids with Trypsin/EDTA prior to flow cytometry analysis; n = 4. (B) Representative images of the differentiation of UCX® spheroids (three-dimensional) into osteoblast-like cells, chondrocyte-like cells, adipocyte-like cells and respective controls (two-dimensional). Cultures were stained with alkaline phosphatase, alcian blue and oil red O, respectively. Scale bar = 100 and 400 μm. [file 13287_2015_82_MOESM1_ESM.pdf]

Additional file 1:

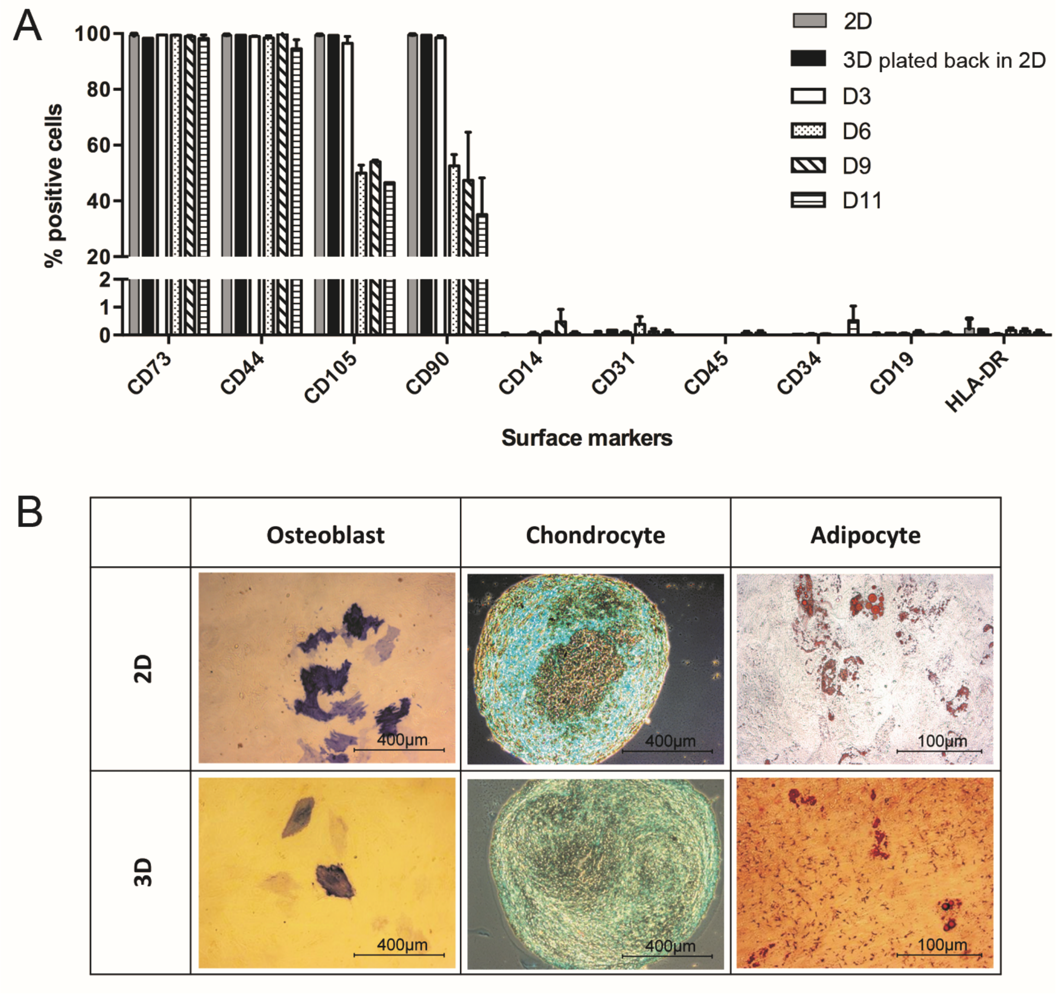

Figure S1 – UCX® spheroids retain the properties of MSCs from adherent cultures. (A) Flow cytometry of surface protein expression on UCX® spheroids at days 3 (D3), 6 (D6), 9 (D9) and 11 (D11) in culture; on UCX® cells collected from spheroids 3D cultures at day 7 and plated back in 2D cultures; and on UCX® originally cultured in 2D conditions. Cells were dissociated from spheroids with Trypsin/EDTA prior to flow cytometry analysis. n = 3. **(B)** Representative images of the differentiation of UCX® spheroids (3D) into osteoblast-like cells, chondrocyte-like cells, adipocyte-like cells and respective controls (2D). Cultures were stained with alkaline phosphatase, alcian blue and oil red O, respectively. Scale bar, 100 and 400 μm.
